# Is the Left Kidney the Right One for Kidney Donation in Women Planning on Future Pregnancy?

**Published:** 2015-11-01

**Authors:** H. Kıran, G. Kıran, D. Arıkan, M. Yüzbaşıoğlu, M. Bakacak, Ö. Ercan, B. Köstü

**Affiliations:** 1 Department of Obstetrics and Gynecology, Kahramanmaras Sutcu Imam University, School of Medicine, Kahramanmaraş, Turkey,; 2 *Department of General Surgery and Transplantation Center, Sanko University School of Medicine, Gaziantep, Turkey*

**Keywords:** Kidney transplantation, Nephrectomy, Pregnancy, Hydronephrosis

## Abstract

The kidney transplantation surgery requires left nephrectomy because of the anatomical disadvantages. But hydroureteronephrosis is the most significant renal functional alteration of pregnancy, accounted for by both hormonal and mechanical factors. Dilatation of the ureters and renal pelvis is more prominent on the right side than the left side and is seen in up to 80% of pregnant women. A 23-year-old woman who become pregnant after 4 months from left kidney donation was admitted to our emergency department with acute right kidney injury during her 39^th^ week of pregnancy. She did not response to conservative treatment and required emergency delivery because of the progressive increase in her serum creatinine levels. After delivery, progressive decrease at creatinine level had been observed and in one month, it had reached the normal level. Mother candidates should be advised they donate their kidneys after completing their childbearing if possible, or undergo right nephrectomy.

## INTRODUCTION

Kidney transplantation, particularly from a living donor, is the treatment of choice for most patients with end-stage renal disease [1]. The superior results achieved with kidney transplantation from living donors have resulted in an increase in this method of transplantation [2]. Renal parenchymal disease includes maladies that damage the outermost internal region of the kidney where filtration and urine formation occur. Vesicoureteral reflux, history of urinary tract infection and hydronephrosis are the most important risk factors for renal parenchymal damage [3-5]. Hydronephrosis or hydroureter is a normal finding in pregnant women. The renal pelvises and caliceal systems may be dilated as a result of progesterone effects and mechanical compression of the ureters at the pelvic brim. Dilatation of the ureters and renal pelvis is more prominent on the right side than the left side and is seen in up to 80% of pregnant women [6]. In most cases, it disappears within a few weeks after delivery. The vast majority of asymptomatic cases are treated conservatively [6]. But, knowledge on perinatal and maternal outcomes in kidney donors are scanty. In this report*, *we present on a woman with a donated left kidney who did not respond to conservative treatment and required emergency delivery because of the progressive increase in her serum creatinine levels.

## CASE REPORT

A G_2_P_0_A_1_ 23-year-old woman who become pregnant after four months of donation was admitted to our emergency department with renal parenchyma damage during her 39^th^ week of pregnancy—serum creatinine rose from 1.2 mg/dL three weeks previously to 2.6 mg/dL. The pre-operative evaluation of the woman who underwent left nephrectomy to donate her kidney to her husband revealed 2/6 HLA matched, HLA-B:51, and HLA-DRB1:14. She had a pre-operative creatinine level of 0.5 mg/dL, BUN of 12.7 mg/dL and blood pressure of 120/80 mm Hg. Her left kidney was 109×36 mm in size and had a mean parenchyma thickness of 12 mm; her right kidney was 101×32 mm and the mean parenchyma thickness was 12 mm. There was no solid or cystic mass; nor was there gynecological pathology at abdominal and pelvic ultrasound examination. The patient came to our Obstetric Outpatient Clinic for the first time at 36^th^ week of her gestation, 12 months after her nephrectomy. Her blood pressure was 100/60 mm Hg. Obstetric ultrasound examination revealed a BPD of 33w+5d, AC of 32w+6d, FL of 32w+4d; the amniotic fluid was enough and the plasenta was at corpus posterior. There was three weeks of intrauterine growth retardation (IUGR) at ultrasound examination according to her last normal menstrual period. Her serum creatinine level was 1.2 mg/dL and BUN was 16.3 mg/dL. Urinalysis showed no sign of infection.

Three weeks after her last follow-up visit, she came to our clinic complaining of decreased urine output and right flank pain. There was three weeks of IUGR at ultrasound at 39^th^ week of gestation; BPD was 36w+3d, FL 36w+4d, and AC 36w+2d. Prepartum renal ultrasound examination revealed that her right kidney was 110×48 mm in size with a mean parenchyma thickness of 17 mm. Parenchyma echogenity was increased (grade 2). There was mild enlargement at pelvicalyxeal system; the pelvic anteroposterior diameter was 15 mm. There was grade 2 renal parenchyma damage and mild hydronephrosis at the right kidney. Because antepartum serum creatinine and BUN levels were increased from 2.1 to 2.6 mg/dL and from 25.8 to 28.5 mg/dL, respectively, we consulted a nephrologist. The Nephrology Department advised emergency delivery because of the progressive increase in creatinine levels. With vaginal delivery a healthy 2500-g girl baby was delivered with an Apgar scores of 9 and 10 for the 1^st^ and 5^th^ minutes. Post-partum blood pressure of the patient was 120/70 mm Hg; her pulse rate was 88/minute. Her post-partum serum creatinine levels at 6^th^, 18^th^, and 42^nd^ hours were 2.7, 2.3, and 1.8 mg/dL, respectively; her BUN levels were 27.5, 25.0, and 26.7 mg/dL, respectively. The patient was discharged on the 3^rd^ day postpartum. One month postpartum, her serum creatinine and BUN levels were measured at 0.85 mg/dL and 13.3 mg/dL, respectively. 

## DISCUSSION

Hydroureteronephrosis is the most significant renal functional alteration during pregnancy, accounted for by both hormonal and mechanical factors. Stasis caused by dextrorotation of the expanding uterus is the principal mechanical factor leading to hydroureteronephrosis of pregnancy and explains its tendency to occur on the right side. There is probability that hydronephrosis during pregnancy develops as a result of compression of the ureters between the pregnant uterus and the *linea terminalis*. It appears that acute hydronephrosis or worsening of an existing hydronephrosis has been somewhat overlooked as a possible cause of uncertain abdominal pain during pregnancy. Dilatation of the ureters and renal pelvis is more prominent on the right side than the left side and is seen in up to 80% of pregnant women. 

The patient came to our clinic complaining of right flank pain. In cases of continued pain or affected renal function, treatment should consist of the insertion of a ureteral catheter [6]. Alternative treatments of symptomatic gestational hydronephrosis include induction of labor and delivery [7], and epidural block [8]. Because of her term pregnancy, she delivered with normal vaginal delivery.

Potential female donors frequently ask whether unilateral nephrectomy will impair future childbearing capabilities, and whether pregnancy negatively affects the kidney donation. Wrenshall, *et al*, concluded that donor nephrectomy was not detrimental to the prenatal course or outcome of future pregnancies [9]. Some potential female donors are anxious whether unilateral nephrectomy will impair the course of pregnancy and fetal growth. Moreover, in a large cohort study, postdonation (*vs* predonation) pregnancies were associated with a lower likelihood of full-term deliveries and a higher likelihood of fetal loss. Postdonation pregnancies were also associated with a higher risk of gestational diabetes, gestational hypertension, proteinuria and pre-eclampsia [10]. Although the hypertension did not exist in our patient, three weeks of IUGR was detected in the fetus.

Counseling about contraception and pregnancy after donation should be initiated during the postdonation evaluation process. Although there is no clear evidence as to the timing of pregnancy postdonation, it is suggested that women should delay pregnancy until at least two months to a year postdonation to allow and assess for the degree of renal compensation and to assess the baseline blood pressures, GFR, and microalbuminuria [11, 12]. A high level of surveillance and monitoring is suggested for all pregnant women postdonation [12]. Our patient became pregnant after four months from donation.

The renal parenchyma damage in our patient with nephrectomized left kidney was thought to have been arisen from hydronephrosis caused by the pressure of the uterus on the ureter. The pregnancy resulted in a healthy baby, although the course of pregnancy was complicated by renal parenchymal damage. As a matter of fact, BUN and creatinine levels returned to normal quickly after the delivery. Even they are asymptomatic, follow up of patients with BUN and creatinine levels would be prudent. 

Kidney transplantation surgery requires left nephrectomy because of the anatomical disadvantages. It is more convenient with respect to mother health to follow nephrectomized pregnant women along with a right nephrectomy. We can advise the candidates who plan to become pregnant to do so after completing childbearing or to have the right kidney removed.

## Notes

**FINANCIAL SUPPORT:** None declared.
